# A Sixty-Hour Week for Mental Hospital Workers

**Published:** 1912-06-01

**Authors:** A. R. Urquhart

**Affiliations:** James Murray's Royal Asylum, Perth.


### THE EDITOR'S LETTER-BOX.

### A Sixty-hour Week for Mental Hospital Workers.

Sir,—Your article in the forefront of The Hospital for May 25 is of great interest to those of us who are responsible for the management of the mental hospitals of the country. Although it would appear that the Royal Asylums of Scotland are not included in the Hours of Work Bill as they were not included in the benefits of the Superannuation Act, still they may be affected, although not directly, by the provisions of any such Act as may be passed, and I shall be glad to have your judgment upon a sixty-hour week for the nurses employed by other hospitals, not only the hospitals and infirmaries in London and great medical centres, but also for the nurses working in small and struggling provincial hospitals, in fever hospitals, in lying-in hospitals, in the innumerable nursing homes which have sprung up in such numbers within these last few years; and I would also desire to be informed what are the hours of work imposed upon nurses in co-operative homes, or occupied on their own account as a matter of professional business. Specially will you differentiate for these nurses the freedom from risk of bodily injury and from the performance of duties which educated physicians and surgeons would describe as *disgusting*. I suggest to you that it would be well that you should investigate the conditions of service in the various parts of the country, and give your advice as to whether the factory system is really appropriate to professional labours.

I gather that you have doubts on this point, although you think that the Scottish Division of the Medico-Psychological Association have somewhat illogically expressed their strong disapproval. However, that Division may well leave it to their English colleagues to take the opposite view, especially in regard to the vast mental hospitals of London and Lancashire; for the conditions in Scotland are so very different that they might even be encouraged to take their own line in formulating their own ideas of nursing administration. It is a far cry to the work of the late Dr. W. A. F. Browne, which he placed on record in a book called "What Asylums Were, Are, and Ought to Be," 1837. From time to time the Scottish Division of the Medico-Psychological Association have given special attention to the requirements of nursing, preparing in 1884 the well-known Handbook for Attendants on the Insane, and in 1900 investigating the conditions of attendants' and nurses' service in every relation.

The result of this investigation was unfortunately considered private, and accessible only to those who furnished the information upon which it was founded. But Scotland has an excellent record for the last generation in raising the efficiency of the nursing service in mental hospitals without any appeal to the powers behind "industrial unrest." Might it not therefore be left to the General Board of Lunacy and the local authorities of Scottish mental hospitals to work out these problems without Parliamentary interference? I might go on to show what mischief is threatened to the interests of patients and staff in Murray's Royal Asylum, and what is the threatened disaster to the poor private insane of the County of Perth. Meanwhile, I content myself by saying that Murray's Asylum has been developed as a small central hospital with small succursal houses, which entail a method of administration by day and by night utterly unsuitable on every ground for the factory system of hours of work.

Yours faithfully,

A. R. Urquhart.

James Murray's Royal Asylum, Perth.
May 27.

